# Hsp90 chaperone code and the tumor suppressor VHL cooperatively regulate the mitotic checkpoint

**DOI:** 10.1007/s12192-021-01240-2

**Published:** 2021-09-29

**Authors:** Mark R. Woodford, Sarah J. Backe, Laura A. Wengert, Diana M. Dunn, Dimitra Bourboulia, Mehdi Mollapour

**Affiliations:** 1 Department of Urology, SUNY Upstate Medical University, 750 East Adams St., Syracuse, NY 13210 USA; 2 Department of Biochemistry and Molecular Biology , SUNY Upstate Medical University, 750 E. Adams St., Syracuse, NY 13210 USA; 3 Upstate Cancer Center, SUNY Upstate Medical University, 750 E. Adams St., Syracuse, NY 13210 USA; 4 Present Address: Department of Biochemistry and Biophysics, School of Medicine and Dentistry, University of Rochester, Rochester, NY USA

**Keywords:** Heat shock protein 90, Chaperone code, Phosphorylation, Kinase, Ubiquitination, Clear cell renal cell carcinoma, Molecular chaperones, Mitotic checkpoint, Mps1, VHL, Von Hippel-Lindau, Tumor suppressor

## Abstract

Heat shock protein-90 (Hsp90) is an essential molecular chaperone in eukaryotes that plays a vital role in protecting and maintaining the functional integrity of deregulated signaling proteins in tumors. We have previously reported that the stability and activity of the mitotic checkpoint kinase Mps1 depend on Hsp90. In turn, Mps1-mediated phosphorylation Hsp90 regulates its chaperone function and is essential for the mitotic arrest. Cdc14-assisted dephosphorylation of Hsp90 is vital for the mitotic exit. Post-translational regulation of Hsp90 function is also known as the Hsp90 “Chaperone Code.” Here, we demonstrate that only the active Mps1 is ubiquitinated on K86, K827, and K848 by the tumor suppressor von Hippel-Lindau (VHL) containing E3 enzyme, in a prolyl hydroxylation-independent manner and degraded in the proteasome. Furthermore, we show that this process regulates cell exit from the mitotic checkpoint. Collectively, our data demonstrates an interplay between the Hsp90 chaperone and VHL degradation machinery in regulating mitosis.

## Introduction

The mitotic checkpoint, also known as mitotic spindle assembly checkpoint, regulates chromosome segregation by arresting cells in metaphase until all chromosomes are correctly aligned. The dual specificity protein kinase, Mps1/TTK, is evolutionarily conserved and the master switch for the mitotic checkpoint (Liu and Winey 2012). Mps1 activity fluctuates during the cell cycle, peaking at early mitosis and abruptly declining during mitotic exit and progression into the G1 phase (Benzi and Piatti 2020; Liu and Winey 2012). High expression and post-translational modification of Mps1 are involved in its activation, whereas the major route of Mps1 inactivation is degradation (Benzi and Piatti 2020; Liu and Winey 2012). Mps1 degrades by the ubiquitin–proteasome pathway in a cell cycle dependent manner through the sequential actions of anaphase promoting complexes–cyclosome (APC–C^Cdc20^ and APC–C^Cdh1^) (Cui et al. 2010). In addition, Ufd2, a U-box-containing ubiquitylation enzyme, is also involved in Mps1 degradation (Liu et al. 2011). Autophosphorylation of serine and threonine residues on Mps1 regulate its kinase activity and its association with centrosomes in mitotic human cells (Thoma et al. 2009). Previous work has shown that Mps1 requires Cdc37, a protein kinase targeting subunit of Hsp90 chaperone complex, for its activity (Schutz et al. 1997).

Heat shock protein-90 (Hsp90) is an essential molecular chaperone in eukaryotes and it is responsible for the maturation, protection, and activation of select proteins referred to as “clients” (Dean and Johnson 2021; Genest et al. 2019; Schopf et al. 2017). The vast majority of Hsp90 clients are protein kinases involved in key signal transduction pathways. Cancer cells rely on the Hsp90 chaperone machinery to protect an array of mutated and over-expressed oncoproteins from misfolding and degradation (Mollapour and Neckers 2012; Neckers et al. 2018). Thus, Hsp90 is a critical facilitator of “oncogene addiction” and cancer cell survival.

Hsp90 chaperone activity is regulated by co-chaperone proteins and PTMs such as phosphorylation, acetylation, ubiquitination, and SUMOylation (Backe et al. 2020; Mayer 2010; Mollapour et al. 2010; Walton-Diaz et al. 2013; Woodford et al. 2016). We have previously identified a conserved threonine residue in the amino-domain of Hsp90 that is phosphorylated by the client serine/threonine kinase Mps1. Hsp90-T115 phosphorylation promotes Mps1 stabilization via strengthened Hsp90-Mps1 interaction and increases Mps1 activity, which contributes to mitotic checkpoint arrest (Woodford et al. 2016). Phosphorylation of this residue also dissociates Hsp90 from the phosphatase Cdc14, a protein whose activity is associated with mitotic checkpoint release. Cdc14 dephosphorylates Hsp90-T115 and this dephosphorylation and subsequent dissociation of Mps1 from Hsp90 allows for release of cells from the mitotic checkpoint (Woodford et al. 2016). In addition to the mitotic checkpoint, Mps1 overexpression is observed in many cancers (Ling et al. 2014; Yen and Kao 2005).

The *VHL* gene is responsible for inherited familial VHL cancer syndrome, and mutations of the *VHL* gene, accompanied by loss of heterozygosity, are also found in 70–80% of sporadic clear cell renal cell carcinoma (ccRCC). VHL forms a multi-protein complex VCB-Cul2 (VHL-Elongin C-Elongin B-Cullin-2) and Rbx1 that acts as a ubiquitin-ligase (E3) and directs proteasome-dependent degradation of target proteins (Kuznetsova et al. 2003). In addition, VHL regulates microtubule stabilization and cell cycle progression (Hergovich et al. 2003; Pause et al. 1998), negatively regulates Mad2 (mitotic arrest deficient 2) protein levels, and maintains chromosomal stability (Hell et al. 2014; Thoma et al. 2009). Phosphorylation of VHL by the mitotic Aurora-A serine/threonine kinase has also been reported (Martin et al. 2013). Here we have shown that Mps1 is subject to ubiquitination and degradation by VHL in an oxygen-independent manner. Our data also reveals that VHL-mediated turnover of Mps1 hastens cell exit from the G2/M checkpoint.

## Materials and methods

### Protein extraction, immunoprecipitation, and immunoblotting

Proteins were extracted from transiently transfected human embryonic kidney (HEK293) as previously described. (Woodford et al. 2016) For immunoprecipitation, cell lysates were incubated with anti-FLAG antibody conjugated beads (Sigma) for 2 h at 4ºC. Immunopellets were washed 3 times with lysis buffer (20 mM HEPES (pH7.0), 100-mM NaCl, 1-mM MgCl_2_, 0.1% NP40, protease inhibitor cocktail (Roche), and PhosSTOP (Roche)). Precipitated proteins were resuspended in 5X Laemmli buffer, boiled, separated by SDS-PAGE, and transferred to nitrocellulose membranes. Co-immunoprecipitated proteins were detected by immunoblotting. Immunoblotting was performed with the indicated antibodies recognizing VHL, HA, HIF1α, human Mps1 (TTK) (Cell Signaling), Ubiquitin (Santa Cruz Biotechnology), Hsp90, GAPDH (ENZO Life Sciences), 6x-His, and FLAG (Thermo Fisher Scientific).

### Bacterial expression and purification of proteins

All proteins were expressed in *E. coli* strain BL21 (DE3) and included an N-terminal 6x-His tag. Purification buffers included 20–50 mM tris or phosphate pH 8.0 and 10 mM β-mercaptoethanol. Chromatography resins were purchased from GE Healthcare Bio-Sciences (Malborough, MA) except for Ni–NTA agarose, which was purchased from Qiagen (Valencia, CA). Transformed cells were grown at 37˚C in LB with 50 mg/L ampicillin until OD_600_ = 0.6. For Hsp90α, and cultures were then cooled to 20˚C and induced with 20 mg/L IPTG overnight. Cells were harvested by centrifugation and lysed enzymatically. Hsp90α expressed in the supernatant and was isolated by sequential Ni–NTA metal affinity (10–250 mM imidazole step gradient), Q-Sepharose anion exchange (0–1 M NaCl gradient), and Superdex-75 size exclusion chromatography. Purified Mps1 and catalytic inactive Mps1-mutant D664A were nucleotide free evidenced by an A_280/260_ ratio of 1.83. Proteins were > 90% pure by SDS-PAGE. Concentrations were determined using calculated extinction coefficients as previously described (Woodford et al. 2017). Proteins were flash frozen on dry ice and stored at − 80˚C until use.

### VHL-mediated ubiquitination of Mps1

50 ng Mps1-His_6_ and its catalytic inactive mutant D664A were bound to Ni–NTA agarose and then incubated with VHL complex (Millipore), containing 25 mM MOPS pH7.5, 0.01% Tween 20, 5 mM MgCl2, 10 μM ATP, 1 ng UBE1 (Millipore), 1 ng UbcH5c (Millipore), and 2 ng GST-ubiquitin. The reaction is initiated with the addition of GST-ubiquitin. After 30 min at 30˚C, the reaction Ni–NTA agarose was washed with lysis buffer. The Ni–NTA agarose was resuspended in 5X Laemmli buffer, boiled, separated by SDS-PAGE, and transferred to nitrocellulose membranes. Ubiquitination was detected with by immunoblotting using anti-ubiquitin antibody.

### Flow cytometry

FACS analysis was performed according the protocol in the Annexin V/FITC kit (Bio-Rad). In brief, following release from nocodazole, cells were trypsinized, collected, and washed once with 1x binding buffer (included in kit). Propidium iodide was added and immediately run on a Becton Dickinson LSRFortessa (BD Biosciences). Data were analyzed using FlowJo software v10.6.2 (BD).

## Results

### Mps1 kinase activity is essential for its VHL-mediated ubiquitination

**Fig. 1:**
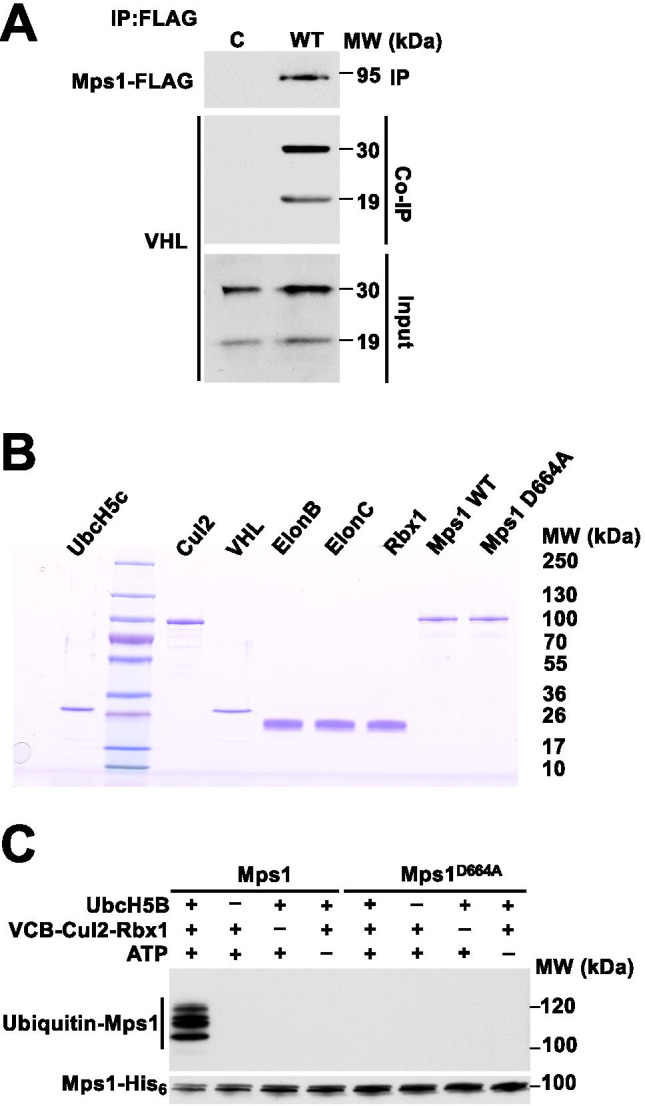
VHL-mediated ubiquitination of the Mps1 kinase. (A) FLAG human Mps1 was transiently expressed and immunoprecipitated from HEK293 cells. Co-immunoprecipitation (Co-IP) of endogenous VHL_30_ and VHL_19_ was assessed by immunoblotting. Empty vector (EV) was used as a control. (B) VHL-elongin C, elongin B, cullin and RBX1 complex (VCB-CR), ubiquitin ligase UbcH5c, human Mps1 and its catalytic inactive mutant D664A were bacterially expressed and purified. 50 ng of each purified protein was resolved on the SDS-PAGE gel and stained with Coomassie Brilliant Blue. (C) Wild-type human Mps1 and the D664A mutant were ubiquitinated in vitro. Total Mps1 was detected by immunoblotting using anti-hexahistidine antibody and ubiquitination with anti-ubiquitin antibody

We have previously shown that Mps1 is upregulated in the *VHL* deficient ccRCC cell line 786-O. In order to determine whether Mps1 is a substrate of VHL, we first established Mps1 interaction with VHL, by transiently expressing and immunoprecipitating Mps1-FLAG in HEK293 cells. The VHL protein is expressed as two isoforms: VHL_30_, a protein of 30 kDa, and VHL_19_, roughly 19 kDa in size (Kim and Kaelin 2004). Both isoforms appear to retain tumor suppressor activity, and for simplicity, the term “VHL” is used when referring to both isoforms generically. Mps1-FLAG interacts with both VHL isoforms (Fig. 1A).

We next obtained further evidence that Mps1 is directly ubiquitinated by VCB-Cul2 complex using in vitro ubiquitination assay kit (Millipore) with the VCB-Cul2 (VHL_30_-Elongin C-Elongin B-Cullin-2) complex. VHL is part of a multi-protein complex, VCB-Cul2 and Rbx1, acting as a ubiquitin-ligase (E3) and directing proteasome-mediated degradation of the substrate proteins. Wild-type Mps1-His_6_ was bacterially expressed, purified (Fig. 1B), and used in our in vitro ubiquitination assay as previously described (Dushukyan et al. 2017). We show that the recombinant wild-type Mps1-His_6_ was subject to ubiquitination (Fig. 1C). Conversely the catalytic inactive recombinant Mps1-D664A-His_6_ mutant was not ubiquitinated (Fig. 1C). Taken together, our data provides a switching off mechanism via ubiquitination and degradation of Mps1 kinase; however this feedback depends on the catalytic activity of Mps1.

### VHL degrades Mps1 in an oxygen-independent manner

**Fig. 2:**
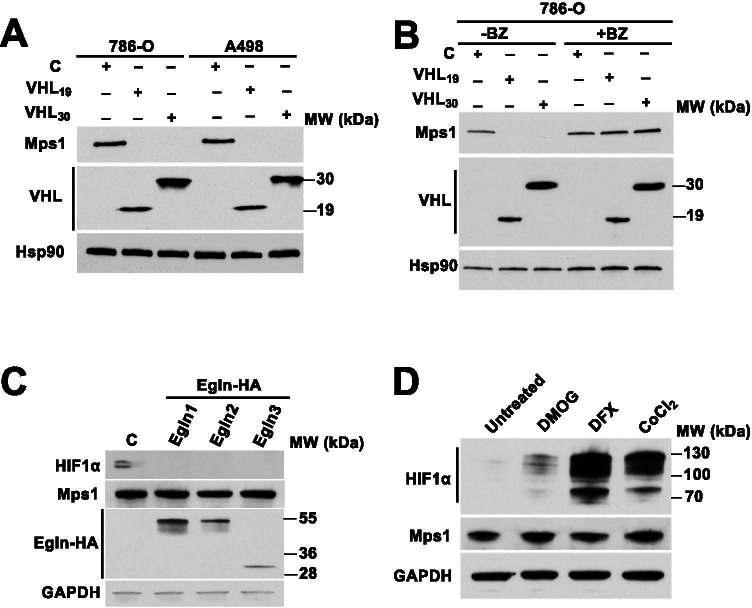
VHL degrades Mps1 in prolyl-hydroxylation independent manner. (A) Mps1 levels were detected by immunoblot in VHL null ccRCC cells 786-O and A498 expressing either VHL_30_ or VHL_19._ (B) VHL null 786-O ccRCC cells expressing either VHL_30_ or VHL_19_ were treated with the proteasome inhibitor Bortezomib (0.5 µM, 4 h). Mps1 levels were detected by immunoblotting with anti-human Mps1 (TTK) antibody. (C) Prolyl-hydroxylases Egln1-HA, Egln2-HA and Egln3-HA were over-expressed in HEK293 cells and Mps1 protein levels were assessed by immunoblotting with anti-Mps1 antibody. (D) HEK293 cells were treated with the prolyl hydroxylase (PHD) inhibitor dimethyloxaloylglycine (DMOG; 500 µM) or the hypoxia mimetic compounds deferoxamine (DFX; 250 µM) or CoCl_2_ (150 µM) for 18 h. Mps1 and HIF1α protein levels were examined by immunoblotting. GAPDH was used as a loading control

Overexpression of either isoform of VHL (VHL_30_-FLAG or VHL_19_-FLAG) in 786-O and A498 cells (VHL null) reduced Mps1 levels (Fig. 2A). This observation is not at the transcriptional level, as the presence or absence of VHL does not affect Mps1 transcription (data not shown). Although expression of both VHL isoforms leads to degradation of Mps1, we were able to prevent this process by pre-treatment with the proteasome inhibitor bortezomib, providing further evidence that VHL-mediated degradation of Mps1 occurs via the proteasome (Fig. 2B). It is very well known that VHL recognizes proline-hydroxylated substrates. We therefore expressed three isoforms of Egg laying 9 (Egln) proteins that catalyze prolyl-hydroxylation. Surprisingly, Mps1 degradation was not observed under these conditions, whereas the canonical VHL substrate HIF1α was destabilized (Fig. 2C). We obtained further evidence by chemically induced hypoxia which led to stabilization of HIF1α; however Mps1 levels were unaffected (Fig. 2D). Taken together, our data demonstrate that both VHL isoforms have the ability to recognize and ubiquitinate Mps1 in an oxygen-independent manner.

### Mps1 ubiquitination regulates cell cycle progression

**Fig. 3:**
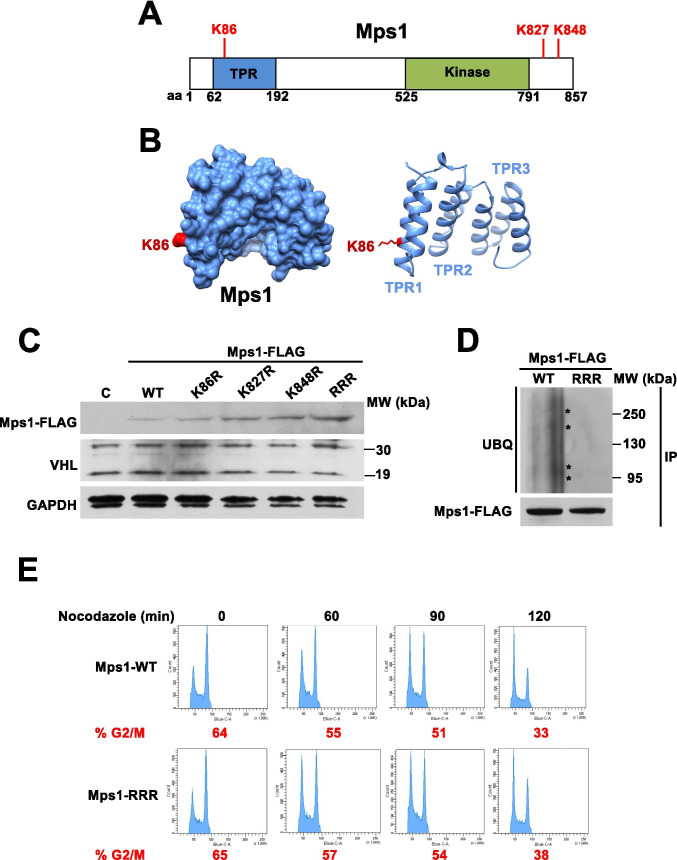
Increased Mps1 stability delays G2/M progression. (A) Schematic of the Mps1 protein with the TPR domain (blue), kinase domain (green), and ubiquitinated residues K86, K827, and K848 highlighted. (B) Structure of the Mps1 TPR domain (PDB ID: 4B94) in blue (Nijenhuis et al. 2013). The ubiquitinated lysine K86 is highlighted in red. (C) Expression of 2 µg wild-type Mps1-FLAG, individual K86R, K827R, K848R, and triple mutant (RRR) in HEK293 cells evaluated by immunoblot using an anti-FLAG antibody. (D) Immunoprecipitation of wild-type Mps1-FLAG or Mps1-RRR-FLAG from HEK293 cells. Ubiquitination of Mps1 was detected using an anti-ubiquitin antibody. Asterisks indicate ubiquitinated-Mps1 bands. (E) HEK293 cells expressing either wild-type Mps1-FLAG or Mps1-RRR-FLAG were synchronized using nocodazole (20 µg/ml). Following release from mitotic arrest, cells were collected at the specified timepoints, stained with propidium iodide, and assayed for cell cycle progression by flow cytometry. The data is representative of three independent experiments

In an attempt to map the lysine (K) sites in Mps1 that are subject to ubiquitination, we screened three residues previously reported to be subject to ubiquitination that are predicted to be important for Hsp90 interaction (Fig. 3A-B; phosphosite.org). We therefore mutated lysine sites to arginine (R) and examined the stability of Mps1 in HEK293 cells. Our data showed K86R, K827R, and K848R mutations stabilized Mps1 (Fig. 3C). The triple mutation K86, 827, and 848R (Mps1-RRR) further stabilized Mps1 (Fig. 3C), and completely abolished Mps1 ubiquitination (Fig. 3D).

Mps1 is a mitotic checkpoint kinase with stability that fluctuates throughout the cell cycle. We therefore examined the impact of RRR triple mutation towards cell cycle regulation. We arrested the cells at G2/M cell cycle checkpoint as a result of nocodazole treatment. Releasing the cells in nocodazole-free medium allowed the WT Mps1-expressing cells to progress through the cell cycle. However, this process was mildly delayed in cells expressing the hyperstable Mps1-RRR mutant (Fig. 3E). Mps1-RRR mildly impacted G2/M progression. However, based on the known role of Mps1 in regulation of G2/M checkpoint, we expect VHL-mediated ubiquitination and degradation of Mps1 to regulate the cell cycle.

## Discussion

**Fig. 4:**
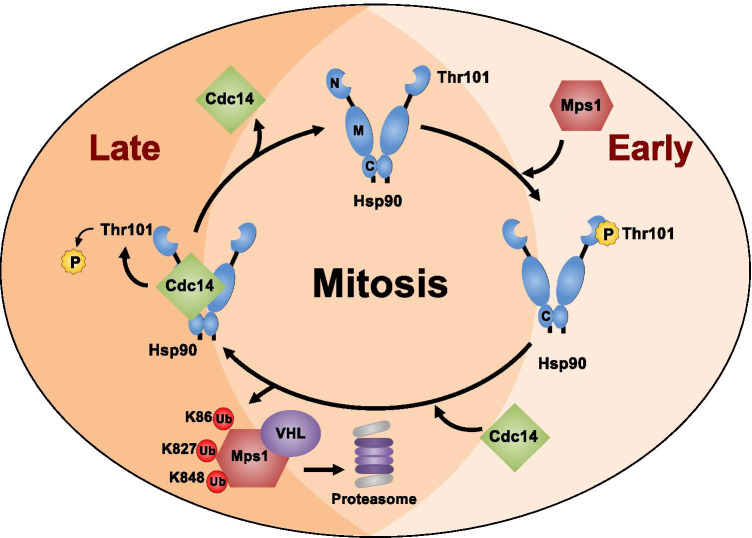
Reciprocal regulatory mechanism between Mps1 and Hsp90. Schematic representation of Mps1-mediated phosphorylation and Cdc14-facilitated dephosphorylation of T115-Hsp90. At early mitosis Mps1 levels and activity increases, therefore it binds and phosphorylates T115-Hsp90 (Woodford et al. 2016). This promotes formation of Hsp90:Mps1 complex. Later in mitosis, Cdc14 dephosphorylates T115-Hsp90, disrupts Hsp90:Mps1 complex, and promotes VHL-mediated Mps1 ubiquitination on K86, K827, and K848 proteasomal degradation. This is important for cells to exit mitosis. Dissociation of Cdc14 allows Mps1 binding to restart the phosphorylation cycle

Post-translational modifications of Hsp90 have been shown to fine tune its chaperone function. This phenomenon is also known as the “chaperone code” (Backe et al. 2020). Our previous work has shown that the evolutionarily conserved dual specificity protein kinase Mps1 phosphorylates a conserved threonine residue (T101 in yeast Hsp90 and T115 in human Hsp90α) in the amino-domain of Hsp90 (Woodford et al. 2016). This in turn regulates the chaperone function by reducing Hsp90 ATPase activity and promoting its association with kinase client proteins including Mps1. We also demonstrated that the Mps1-mediated phosphorylation of Hsp90 is essential for the mitotic checkpoint because it leads to both stability and activity of Mps1 kinase (Woodford et al. 2016). We further showed Cdc14 as the phosphatase that dephosphorylates T101 and disrupts Msp1-Hsp90 complex. Consequently, this leads to Mps1 degradation, providing a unique regulatory mechanism for its inactivation and facilitating the exit from mitosis (Fig. 4) (Woodford et al. 2016).

In this study we show that the tumor suppressor VHL, the substrate recognition subunit of an E3 ligase, is involved in ubiquitination and degradation of Mps1 (Fig. 4). This process depends on Mps1 catalytic activity, as the catalytically inactive Mps1-D664A is not ubiquitinated (Fig. 1C). Previous works have shown that APC-C and the U-box-containing ubiquitination enzyme Ufd2 are also involved in degradation of Mps1 (Cui et al. 2010; Liu et al. 2011). Therefore it is important to delineate the roles of VHL, APC-C, and Ufd2 in ubiquitination of Mps1 and regulation of the mitotic checkpoint. Collectively, our findings show that Hsp90 and VHL protect and degrade Mps1, respectively, consequently regulating the mitotic checkpoint.
